# Cytotoxin-Associated Gene A-Negative *Helicobacter pylori* Promotes Gastric Mucosal CX3CR1^+^CD4^+^ Effector Memory T Cell Recruitment in Mice

**DOI:** 10.3389/fmicb.2022.813774

**Published:** 2022-01-27

**Authors:** Heqiang Sun, Taojun He, Yanan Wu, Hanmei Yuan, Jie Ning, Zhenhua Zhang, Xinli Deng, Bin Li, Chao Wu

**Affiliations:** ^1^Department of Laboratory Medicine, The Second Medical Center & National Clinical Research Center for Geriatric Diseases, Chinese PLA General Hospital, Beijing, China; ^2^Department of Laboratory Medicine, The Eighth Affiliated Hospital of Sun Yat-sen University, Shenzhen, China; ^3^Peking University People’s Hospital, Peking University Institute of Hematology, Beijing Key Laboratory of Hematopoietic Stem Cell Transplantation, Beijing, China; ^4^Department of Gastroenterology of the 305 Hospital of Chinese People’s Liberation Army, Beijing, China

**Keywords:** *Helicobacter pylori*, CagA, effector memory T cell, CX3CL1, CX3CR1

## Abstract

**Background:**

*Helicobacter pylori* can cause many kinds of gastric disorders, ranging from gastritis to gastric cancer. Cytotoxin-associated gene A (CagA)^+^*H. pylori* is more likely to cause gastric histopathologic damage than CagA^–^*H. pylori*. However, the underlying mechanism needs to be further investigated.

**Materials and methods:**

Mice were intragastrically administered equal amounts of CagA^+^ or CagA^–^*H. pylori*. Four weeks later, 24 chemokines in stomachs were measured using a mouse chemokine array, and the phenotypes of the recruited gastric CD4^+^ T cells were analyzed. The migration pathway was evaluated. Finally, the correlation between each pair among the recruited CD4^+^ T cell sub-population, *H. pylori* colonization level, and histopathologic damage score were determined by Pearson correlation analysis.

**Results:**

The concentration of chemokines, CCL3 and CX3CL1, were significantly elevated in CagA^–^*H. pylori*-infected gastric mucosa than in CagA^+^*H. pylori*-infected gastric mucosa. Among them, CX3CL1 secreted by gastric epithelial cells, which was elicited more effectively by CagA^–^*H. pylori* than by the CagA^+^ strain, dramatically promoted mucosal CD4^+^ T cell migration. The expression of CX3CR1, the only known receptor of CX3CL1, was upregulated on the surface of gastric CD4^+^ T cells in CagA^–^*H. pylori*-infected stomach. In addition, most of the CX3CR1-positive gastric CD4^+^ T cells were CD44^+^CD69^–^CCR7^–^ effector memory T cells (Tem). Pearson correlation analysis showed that the recruited CX3CR1^+^CD4^+^ Tem cell population was negatively correlated with *H. pylori* colonization level and histopathologic damage score.

**Conclusion:**

CagA^–^*H. pylori* promotes gastric mucosal CX3CR1^+^CD4^+^ Tem recruitment in mice.

## Introduction

*Helicobacter pylori* is a spiral-shaped, Gram-negative bacterium that resides in the gastric mucosa. It can cause many types of gastric disorders, such as gastritis, gastric ulcers, gastric mucosa-associated lymphoid tissue lymphoma, and gastric cancer. *H. pylori* eradication is effective in the prevention of metachronous gastric cancer (Ernst and Gold, 2000). However, the immune responses elicited by *H. pylori* infection are insufficient for its eradication; therefore, *H. pylori* can persist in the stomach for decades or even life (Hooi et al., 2017; Hu et al., 2017; Crowe, 2019). Cytotoxin-associated gene A (CagA) is an essential virulence factor in *H. pylori* that can be injected into host cells through the type IV secretion system (T4SS) (Odenbreit et al., 2000; Rieder et al., 2005; Frick-Cheng et al., 2016). Compared with CagA^–^*H. pylori*, CagA^+^*H. pylori* is more likely to cause gastric histopathologic damage and gastric disorders (van Doorn et al., 1998; Sayehmiri et al., 2015; Yong et al., 2015). CagA^–^*H. pylori* is less strongly linked to malignancy and may only be associated with diffuse types of the disease (Parsonnet et al., 1997). These histopathologic damages may be associated with factors, such as the number and invasiveness of *H. pylori*, corresponding immune responses, and HLA variety in the patients, among others (Kusters et al., 2006). These factors also interact with each other. Here, we focused our attention on the immune responses stimulated by *H. pylori*.

After *H. pylori* infection, CD4^+^ T cell responses are elicited, including T helper 1 cells (Th1, CD3^+^CD4^+^IFN-γ^+^) (Robinson et al., 2007), Th2 cells (CD3^+^CD4^+^IL4^+^) (Larussa et al., 2015), Th17 cells (CD3^+^CD4^+^IL17A^+^) (Dixon et al., 2019), and regulatory T cells (Treg; CD3^+^CD4^+^CD25^+^Foxp3^+^) (Rad et al., 2006), among others. Th1, Th2, and Th17 cells act as effector lymphocytes. Treg cells can inhibit effector lymphocyte responses. Although many studies on vaccination have indicated that effector CD4^+^ T cells play essential roles in protection against *H. pylori* infection, the roles of CD4^+^ T cells, particularly memory T cells, during chronic infection remain unclear (Ermak et al., 1998; Amedei et al., 2006; Chen et al., 2013). Memory CD4^+^ T cells are a type of cell with long-term function, which is of great significance in repeated responses to pathogen infection. Based on their phenotypes, memory T cells are primarily divided into three populations: central memory T cells (Tcm; CD44^+^CD62L^+^CCR7^+^CD127^+^CD69^–^CD103^–^), which patrol secondary lymphoid organs similar to naïve T cells and undergo rapid and robust proliferation, differentiation, and migration to the site of infection; effector memory T cells (Tem; CD44^+^CD62L^–^CCR7^–^CD127^+^CD69^–^CD103^–^), which recirculate between blood and non-lymphoid tissues and execute their effector functions rapidly similar to freshly stimulated effector T cells; and tissue-resident memory T cells (Trm; CD44^+^CD62L^–^CCR7^–^CD11a^+^CD69^+^CD103^+^), which are derived from precursors that enter the tissue during the immune effector phase and remain therein (Mueller et al., 2013; Spitaels et al., 2016). Thus, weak responses of memory T cells may account for the long-term persistence of *H. pylori* in the gastric mucosa.

Chemokines play an essential role in T cells recruitment (Yamaoka et al., 1998; Winter et al., 2010). They can be secreted by many types of cells, such as gastric epithelial cells, macrophages, dendritic cells, neutrophils, B cells, and NK cells, among others (Shimada and Terano, 1998; Bhattacharyya et al., 2002). CD4^+^ T cells expressing corresponding receptors can migrate toward chemokines. In *H. pylori* infection, the migration of CD4^+^ T cells to the gastric mucosa and the corresponding influence of CagA have not been well researched. To answer this question, we investigated the chemotaxis pathway through which CD4^+^ T cells migrate to *H. pylori*-infected gastric mucosa and the influence of CagA in a mouse model of *H. pylori* infection. The data demonstrated that CagA weakened the recruitment of protective gastric mucosal CX3CR^+^CD4^+^ Tem cells in *H. pylori*-infected mice. Our research provides new insights into the mechanism underlying the long-term colonization of *H. pylori* in the gastric mucosa and potential subsequent injury.

## Materials and Methods

### Bacteria and Mice

*Helicobacter pylori* strain NCTC11637 (GenBank accession number: AF202973, CagA^+^) and its isogenic mutant ΔcagA NCTC11637 (CagA^–^) were used in this study (Asahi et al., 2000; Suzuki et al., 2009; Li et al., 2017). They were cultured on brain–heart infusion agar containing 10% rabbit blood at 37°C under microaerobic conditions. Two days later, *H. pylori* strains were transferred from the plates to the Brucella broth containing 10% fetal bovine serum (FBS) for active culture. The actively growing *H. pylori* strains were harvested for infection and *in vitro* experiments. To develop a mouse model of *H. pylori* infection, 6–8-week-old female BALB/c mice were infected with *H. pylori* strain NCTC11637 (CagA^+^) or its isogenic mutant ΔcagA NCTC11637 (CagA^–^) through intragastric administration of 1 × 10^9^ CFUs per mouse, once a day for 4 days. In the control group, PBS was used instead of *H. pylori.* Four weeks later, the mice were subjected to analyses. All mice were purchased from the Experimental Animal Center, Third Military Medical University and housed under specific pathogen-free (SPF) conditions. Animal maintenance and experimental procedures were performed in accordance with the National Institutes of Health Guidelines for the Use of Experimental Animals and approved by the Medicine Animal Care Committee of the Third Military Medical University.

### Cell Lines

Gastric epithelial cell line, AGS (Zhu et al., 2017; Yang et al., 2021), was obtained from the American Type Culture Collection (Manassas, VA, United States) and BGC-823 (Tang et al., 2013; Guo et al., 2020) was obtained from the Shanghai Institute CellBank, Chinese Academy of Sciences. The two cell lines were thawed and cultured in 3 ml of the complete Ham’s F12 medium (1 × 10^5^ cells/well) in a 12-well plate. The complete Ham’s F12 medium consisted of the Ham’s F12 medium, 25 mmol/l hydroxyethyl piperazine ethanesulfonic acid (HEPES) (pH 7.2), 100 U/ml penicillin, 100 μg/ml streptomycin, and 10% FBS. After culturing for 1 day, gastric epithelial cells were transferred to new wells and were ready for use.

### Isolation and Immunophenotyping of Gastric Lymphocytes

Gastric lymphocytes were isolated and immunophenotyped as previously described (Ruiz et al., 2012; Sun et al., 2018). In brief, stomachs were washed twice gently with PBS and cut into pieces with sharp scissors. The stomach pieces were then placed in 10 ml of Hank’s balanced salt solution (without Ca^2+^, My^2+^) with 1 mM dithiothreitol, 1 mM ethylenediaminetetraacetate, and 2% fetal calf serum and incubated for 45 min at 37°C with gentle agitation. The mixture was passed through a steel mesh to remove the undigested tissue. The digested single-cell suspensions were harvested and washed twice with PBS. Single cells were stained with BV510-labeled anti-mouse CD45, APC-labeled anti-mouse CD3, APC/Cy7-labeled anti-mouse CD4, PerCP/Cy5.5-labeled anti-mouse CX3CR1, PE/Cy7-labeled anti-mouse CD44, BV421-labeled anti-mouse CD69, and PE-labeled anti-mouse CCR7 antibodies and measured by flow cytometry.

### Quantitative Real-Time PCR

*Helicobacter pylori* colonization in the stomach was detected by quantitative real-time PCR. Bacterial genome was extracted from the mouse stomach using a TIANamp Bacteria DNA Kit (TIANGEN, DP302). Then, *H. pylori* 16S rDNA copies were measured (sense primer, 5′-TTTGTTAGAGAAGATAATGACGGTATCTAAC-3′; anti-sense primer, 5′-CATAGGATTTCACACCTGACTGACTATC-3′; and *H. pylori* 16S probe, FAM-CGTGCCAGCAGCCGCGGT-TAMRA). For quantitative real-time PCR, the target gene with a concentration gradient was set as the standard in addition to positive and negative controls. Two replicates were used for each sample. The number of 16S rDNA copies of *H. pylori* were considered to depict *H. pylori* colonization levels in the mouse stomach, and data were presented as log_10_ values. To measure IFN-γ, IL-17A, and IL-4 responses of CX3CR1^+^CD4^+^ T cells in the mouse stomach, CX3CR1^+^CD4^+^ T cells were sorted by FACS; then total RNA was extracted using TRIzol and reverse-transcribed into cDNA using a PrimeScript TM 1st Strand cDNA Synthesis Kit (Takara). The expression levels of IFN-γ, IL-17A, and IL-4 were determined using quantitative real-time PCR with SYBR green staining. β-Actin was used as the reference gene (IFN-γ sense primer, 5′- GATCCTTTGGACCCTCTGACTT-3′; IFN-γ anti-sense primer, 5′-TGACTGTGCCGTGGCAGTAA-3′; IL-17A sense primer, 5′-CTCCAGAAGGCCCTCAGACTAC-3′; IL-17A anti-sense primer, 5′-GGGTCTTCATTGCGGTGG-3′; IL-4 sense primer, 5′ -GAGCTGCAGAGACTCTTTCG-3′; IL-4 anti-sense primer, 5′-ACTCATTCATGGTGCAGCTTA-3′; β-actin sense primer, 5′-CCTGCAGAGTTAAGCATGCCAG-3′; and β-actin anti-sense primer, 5′-TGCTTGATCACATGTCTCGATCC-3′).

### Histological Evaluation

The longitudinal strips of the stomach from *H. pylori*-infected or wild-type (WT) uninfected mice were formalin-fixed, embedded in paraffin, sectioned at 5 μm, and stained with hematoxylin and eosin. Histological evaluation was performed by two experienced pathologists in a blinded fashion at the Department of Pathology, Southwest Hospital, Third Military Medical University. The gastric histopathologic damage score was graded on a scale of 0–5 as previously reported (Garhart et al., 2002). The infiltrates of inflammatory cells, epithelial hyperplasia, and mucous cell metaplasia were included in the histological evaluation.

### Chemokine Array Measurement

Mice were infected with *H. pylori* strain NCTC11637 (CagA^+^) or its isogenic mutant ΔcagA NCTC11637 (CagA^–^) by intragastric administration. Four weeks later, the stomachs were harvested, washed twice gently with PBS, cut into pieces, homogenized, and digested in a cell lysis buffer containing a protease inhibitor cocktail for 30 min. After centrifugation (12,000 rpm, 15 min, 4°C), the supernatant was collected for chemokine array detection using Quantibody^®^ Mouse Chemokine Array 1 (Cat# QAM-CHE-1, RayBiotech, Inc., Guangzhou, China) following the manufacturer’s instructions. In brief, completely air-dried glass chips were blocked with a sample diluent. After washing, 100 μl of samples were added to each well and incubated at room temperature for 2 h. The array was washed again and incubated with detection antibody cocktail for 1.5 h. The chips were washed, followed by incubation with Cy3 equivalent dye–streptavidin for 1 h at room temperature. An InnoScan 300 Microarray Scanner was used to collect fluorescence intensities.

In gastric epithelial cells and *H. pylori* co-culture chemokine secretion experiments, gastric epithelial cell lines AGS and BGC823 were cultured in the complete Ham’s F12 medium for 1 day in a 37°C incubator with 5% CO_2_. Then, gastric epithelial cells were transferred to new wells, 1 × 10^5^ cells/well, and 1 × 10^7^ bacteria/well of CagA^+^*H. pylori*, CagA^–^*H. pylori*, or equal volumes of PBS were added (gastric epithelial cells: *H. pylori* = 1:100). After 24 h, the supernatant was collected for chemokine array measurement following the manufacturer’s instructions (Human Chemokine Array 1, Cat# AAH-CHE-G1, RayBiotech, Inc., Guangzhou, China). An InnoScan 300 Microarray Scanner was used to measure fluorescence intensities.

### Transwell Migration Assay

CD4^+^ T cells were sorted using flow cytometry from the mouse spleen or stomach. Then, sorted 1.0 × 10^5^ CD4^+^ T cells/well were added to the upper chambers of a 24-well transwell plate. The lower chambers were filled with the complete RPMI-1640 medium containing 500 ng/ml of mouse chemokines, CCL-3 and CX3CL1, or PBS as the control. After incubation for 5 h at 37°C in a 5% CO_2_ incubator, the cell number in the lower chambers was counted by flow cytometry. In some cases, the blocking antibody for CX3CL1 (20 μg/ml) or the corresponding control isotype (20 μg/ml) were added into the lower chambers, and anti-CX3CR1 antibody (20 μg/ml) or corresponding control isotype (20 μg/ml) were added to the cell suspensions for 2 h and then added to the upper chambers of the plate for the transwell migration assay. Triplicate wells were established for each condition. Data represent the chemotactic index (number of cells that migrated to the chemokines divided by number of cells that migrated to the complete RPMI-1640 medium plus PBS). The complete RPMI-1640 medium consisted RPMI-1640 medium, 25 mmol/l HEPES (pH 7.2), 100 U/ml penicillin, 100 μg/ml streptomycin, and 10% FBS.

### Statistical Analysis

Differences between the two groups were analyzed using independent-sample *t*-tests. Comparisons of three or more groups and the expression levels of each chemokine in the array data were analyzed by one-way ANOVA with the Bonferroni post-test. Pearson correlation analysis was used to analyze the correlation between the groups. Statistical analysis was performed using SPSS16 software. Differences were considered significant at *p* < *0.05*.

## Results

### CagA^+^ *Helicobacter pylori* Induced Stronger Inflammation but Less Mucosal CD4^+^ T Cell Formation Than CagA^–^ Strain

**FIGURE 1:**
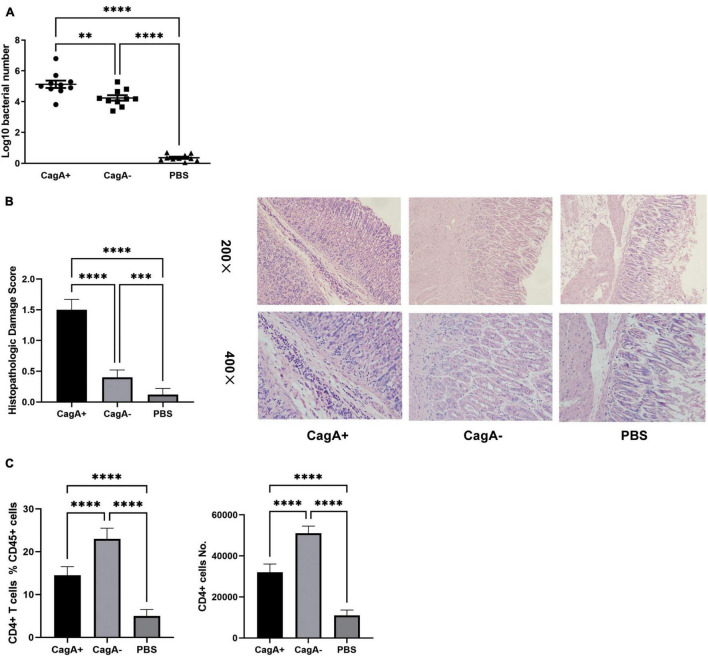
CagA^+^*H. pylori* induced stronger inflammation but less mucosal CD4^+^ T cell formation than CagA^–^ strain. Six- to eight-weeks old mice were infected with *H. pylori* strain NCTC11637 (CagA^+^) or its isogenic mutant ΔcagA NCTC11637 (CagA^–^) *via* intragastric administration with 10^9^ colony-forming units (CFUs) per mouse once daily for 4 days. In the control group, PBS was used instead of *H. pylori.* **(A)** Four weeks later, *H. pylori* colonization in the stomachs were detected by quantitative real-time PCR. **(B)** Mice stomach histopathologic damage score and representative histological images are shown. **(C)** The percentage and number of mucosal CD4^+^ T cells were measured by flow cytometry. Data are shown as mean ± SD. *N* = 10. ^**^p < 0.01, ^***^p < 0.001, and ^****^p < 0.0001. Results are representative of three individual experiments.

Mice were infected with the *H. pylori* strain NCTC11637 (CagA^+^) or its isogenic mutant ΔcagA NCTC11637 (CagA^–^) *via* intragastric administration. In the control group, PBS was used instead of *H. pylori.* Four weeks later, *H. pylori* colonization in the stomach was detected by quantitative real-time PCR. Histopathologic damages in the stomachs were scored, and the percentage and number of CD4^+^ T cells in the gastric mucosa were measured by flow cytometry. The data showed that the colonization of *H. pylori* was significantly higher, accompanied with more severe histopathological damage in the stomach mucosa of CagA^+^*H. pylori*-infected mice than in the stomach mucosa of CagA^–^*H. pylori*-infected mice (Figures 1A,B), although an equal number of CFUs were used for infection *via* intragastric administration. Although the CagA^+^ group showed a larger CD4^+^ T cell population in the gastric mucosa than the PBS group, gastric mucosal CD4^+^ T cell population in the CagA^–^ group was dramatically higher than that in the CagA^+^ group (Figure 1C). Based on these data, we found that CagA affects mucosal CD4^+^ T cell numbers after *H. pylori* infection.

### Expression Level of Gastric Chemokine CX3CL1 and the Number of CX3CR1^+^CD4^+^ T Cells Were Elevated in CagA^–^ *Helicobacter pylori*-Infected Mice

**FIGURE 2:**
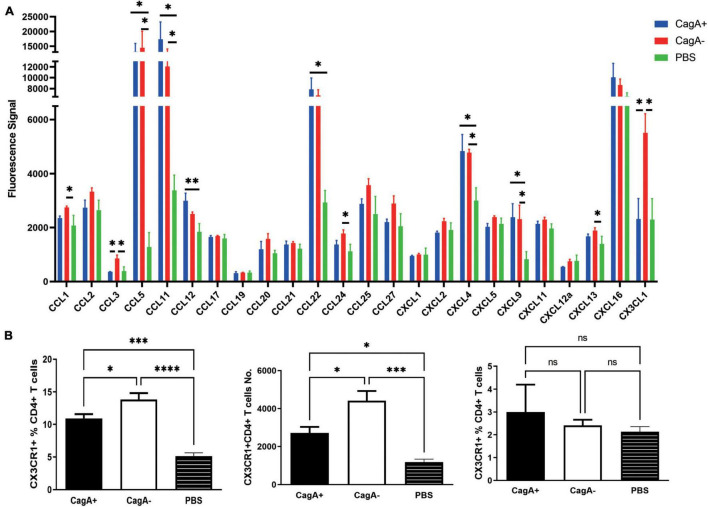
Gastric chemokine CX3CL1 expression and the number of CX3CR1^+^CD4^+^ T cells were significantly elevated in CagA^–^*H. pylori*-infected mice. Mice were infected with *H. pylori* strain NCTC11637 (CagA^+^) or its isogenic mutant ΔcagA NCTC11637 (CagA^–^) *via* intragastric administration. Four weeks later, the stomachs and spleens were harvested. **(A)** A series of mouse mucosal chemokines were measured. **(B)** The percentage of CX3CR1^+^ cells in gastric mucosal CD4^+^ T cells and the number of CX3CR1^+^CD4^+^ T cells were detected (left and middle). The percentage of splenic CX3CR1^+^CD4^+^ T cells in CD4^+^ T cells is also shown (right). Data are shown as mean ± SD. *N* = 5, *p < 0.05, ^**^p < 0.01, ^***^p < 0.001, and ^****^p < 0.0001. Data in **(A)** are representative of two similar experiments. Data in **(B)** are representative of three individual experiments.

To investigate whether chemokines were involved in the difference in the gastric mucosal CD4^+^ T cell population between CagA^+^*H. pylori* and CagA^–^*H. pylori*-infected mice, stomach tissue was harvested from the CagA^+^ and CagA^–^ groups 4 weeks after *H. pylori* infection. A series of mouse chemokines were measured in the stomach using Quantibody^®^ Mouse Chemokine Array 1. Several chemokines increased significantly after *H. pylori* infection compared to that in the PBS control (Figure 2A). CCL3 and CX3CL1 showed significant differences between the CagA^+^ and CagA^–^ groups (Figure 2A). Moreover, the expression of CX3CL1 was much higher than that of CCL3. Next, CX3CR1, the only known CX3CL1 receptor on CD4^+^ T cell surface, was measured by flow cytometry. In the stomach mucosa of mice infected with CagA^–^*H. pylori*, the CX3CR1^+^CD4^+^ T cell population percentage and number were significantly elevated compared with the stomach mucosa of mice infected with CagA^+^*H. pylori* (Figure 2B, left and middle). Spleen CD4^+^ T cells showed no significant difference in CX3CR1 expression between the CagA^+^ and CagA^–^ groups (Figure 2B, right). In addition to CX3CR1, the expression of another 10 chemokine receptors on mucosal CD4^+^ T cells was detected (Supplementary Figure 1). Two of the 11 measured chemokine receptors, CX3CR1 and CXCR4 showed increased expression levels in CagA^–^*H. pylori*-infected mice compared with those in CagA^+^ *H. pylori*-infected mice. But the percentage of CXCR4^+^CD4^+^ T cells (around 0.2–0.5%) was very low compared with CX3CR1^+^CD4^+^ T cells (around 12–14%). Thus, we focused our attention on CX3CR1^+^CD4^+^ T cells and concluded that CagA primarily affected the percentage of mucosal CX3CR1^+^CD4^+^ T cells.

### CX3CL1 Secreted by Gastric Epithelial Cells Was Elicited More by CagA^–^ *Helicobacter pylori* Than by CagA^+^ *Helicobacter pylori*

**FIGURE 3:**
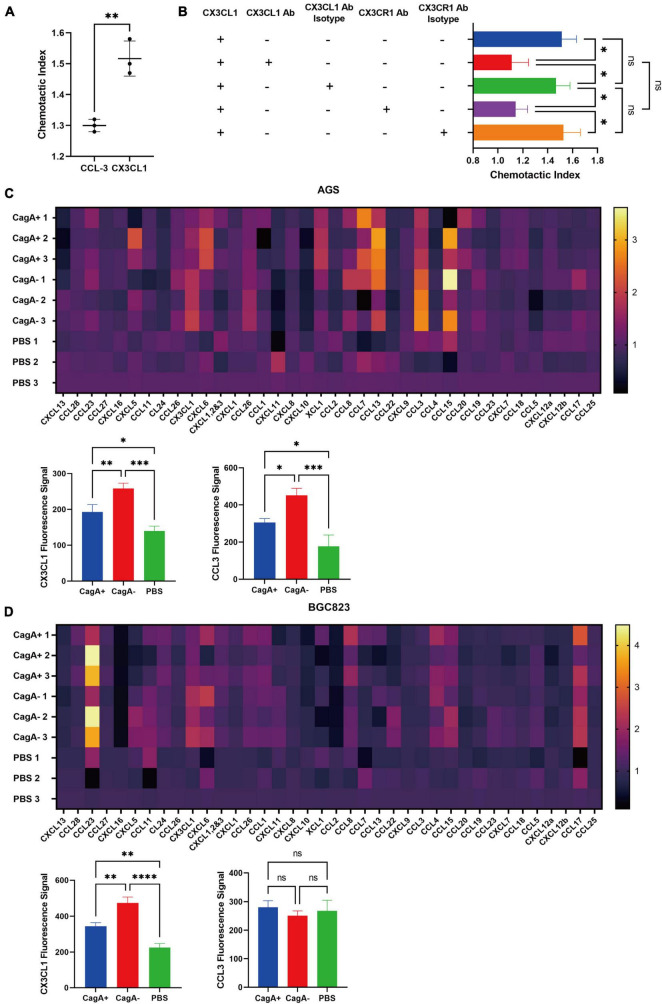
Chemotaxis assay and chemokines secreted by gastric epithelial cell lines. **(A)** Transwell migration assay. Purified splenic CD4^+^ T cells from WT mice were added to the upper chambers of a transwell plate. The lower chambers were filled with the complete RPMI-1640 medium containing 500 ng/ml of mouse chemokine CCL-3, CX3CL1, or PBS as the control. Data are shown as the chemotactic index (number of cells that migrated to the medium containing chemokine divided by the number of cells that migrated to the medium containing PBS). **(B)** Gastric CD4^+^ T cells were isolated from stomachs of CagA^–^*H. pylori*-infected mice, and their migration was assessed *via* transwell assays. Chemotactic indices are shown. **(C,D)** Gastric epithelial cell lines AGS and BGC823were co-cultured with active *H. pylori* strain NCTC11637(CagA^+^) or its isogenic mutant ΔcagA NCTC11637 (CagA^–^) at a ratio of 1:100 (gastric epithelial cells: *H. pylori*) for 24 h. PBS was used as the control. Then, chemokines were detected in the supernatant. Heat maps showed the increased percentage of each chemokine in the *H. pylori* stimulated medium compared with that in the medium containing PBS. The fluorescence signals of CX3CL1 and CCL3 are shown separately. Data are shown as mean ± SD. *N* = 3, *p < 0.05, ^**^p < 0.01, ^***^p < 0.001, and ^****^p < 0.0001. The results in **(A)** are representative of four similar experiments. The results of **(B–D)** are representative of two similar experiments.

To further study whether CD4^+^ T cells could migrate toward CX3CL1, a transwell migration assay was performed. Purified splenic CD4^+^ T cells from WT mice were added to the upper chambers of a transwell plate. The lower chambers were filled with the complete RPMI-1640 medium supplemented with chemokines CCL-3 or CX3CL1, which were significantly increased in CagA^–^*H. pylori*-infected mouse stomachs compared with in the stomach of CagA^+^*H. pylori*-infected mice. PBS was used as the control. Even though the data in Figure 2 drew our attention on CX3CR1, which was the only known chemokine receptor of CX3CL1, to be rigorous, we selected another increased chemokine as control and CCL-3 was the most distinguished one with nearly twofold increase in CagA^–^ group than CagA^+^ group. As a result, CX3CL1 was found to be a more powerful chemokine for mouse CD4^+^ T cell migration (Figure 3A). Subsequently, gastric mucosal CD4^+^ T cells from CagA^–^*H. pylori*-infected mice were harvested for the transwell assay. We found that CX3CR1 and CX3CL1 antibodies inhibited the migration of mucosal CD4^+^ T cells (Figure 3B). Thus, our data confirmed that the CX3CL1–CX3CR1 axis contributes to the accumulation of mucosal CD4^+^ T cells after *H. pylori* infection.

In *H. pylori*-infected mice, gastric epithelial cells are the primary cells that bacteria interact with. In addition, gastric epithelial cells can produce many types of chemokines (Sieveking et al., 2004). To investigate whether gastric epithelial cells could produce CX3CL1 and the influence of CagA^+^ and CagA^–^*H. pylori*, we cocultured the gastric epithelial cell lines, AGS and BGC823, with *H. pylori* strain NCTC11637 (CagA^+^) or its isogenic mutant ΔcagA NCTC11637 (CagA^–^). PBS was used as the control. A series of chemokines were measured in the supernatant. As a result, the concentration of CX3CL1 in the CagA^–^ group was higher than that in the CagA^+^ group for both epithelial cell lines, AGS and BGC823 (Figures 3C,D). CCL3 levels increased in the AGS cell line but not in BCG823 after CagA^–^*H. pylori* stimulation (Figures 3C,D), which indicated different gastric epithelial cell lines would affect CCL3 expression stimulated by H. *pylori*. But as proved above, high CCL3 expression was not related to more recruitment of CX3CR1^+^CD4^+^ T cells in gastric mucosa. Therefore, we concluded that CagA can affect the expression of CX3CL1 by gastric epithelial cells, which plays important role in CX3CR1^+^CD4^+^ T cells recruitment.

### Gastric CX3CR1^+^CD4^+^ T Cells in *Helicobacter pylori*-Infected Mice Were Primarily Effector Memory T Cells

**FIGURE 4:**
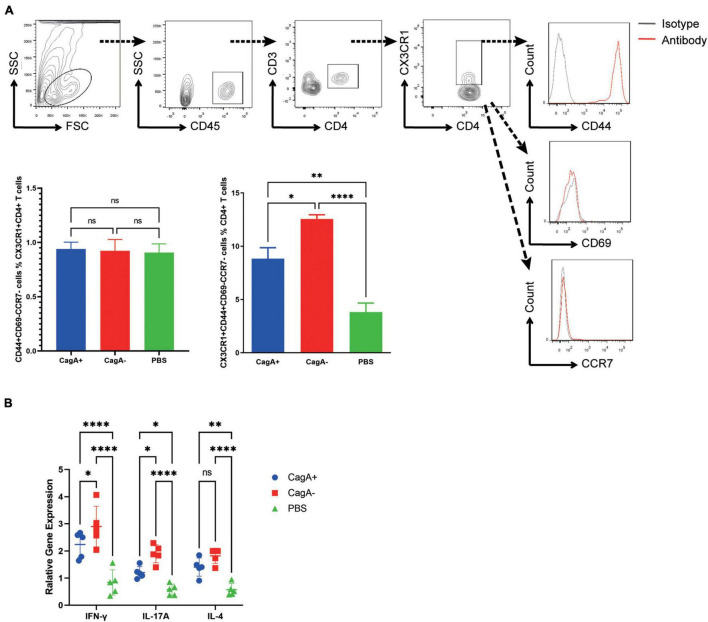
Phenotype of mucosal CX3CR1^+^CD4^+^ T cells in *H. pylori*-infected mice. Mice were infected with *H. pylori* strain NCTC11637 (CagA^+^) or its isogenic mutant ΔcagA NCTC11637 (CagA^–^) by intragastric administration. PBS was used as the control. Four weeks later, stomachs were harvested. **(A)** The expression of CD45, CD3, CD4, CX3CR1, CD44, CD69, and CCR7 in gastric cells was assessed. The percentages of CD44^+^CD69^–^CCR7^–^ population in gastric CX3CR1^+^CD4^+^ T cells and percentages of CD3^+^CD4^+^CX3CR1^+^CD44^+^CD69^–^CCR7^–^ population in gastric CD4^+^ T cells are shown. **(B)** The expression levels of IFN-γ, IL-17A, and IL-4 in sorted CX3CR1^+^CD4^+^ T cells from *H. pylori*-infected mice stomachs were determined by quantitative real-time PCR. β-actin was used as the reference gene. Data are shown as mean ± SD. *N* = 5, *p < 0.05, ^**^p < 0.01, and ^****^p < 0.0001. Results are representative of four individual experiments.

To investigate the phenotype of CD4^+^ T cells migrating to the stomach through the CX3CL1/CX3CR1 pathway, gastric lymphocytes were isolated at 4 weeks post CagA^+^ or CagA^–^*H. pylori* infection, and cell surface markers were detected. We found that the mucosal CD3^+^CD4^+^CX3CR1^+^ cell population from *H. pylori*-infected mice or PBS controls primarily showed a phenotype of CD44^+^, CD69^–^, and CCR7^–^, which were regarded as Tem (Figure 4A). The percentage of CD3^+^CD4^+^CX3CR1^+^CD44^+^CD69^–^CCR7^–^ population (CX3CR1^+^CD4^+^ Tem) in mouse gastric CD4^+^ T cells was higher in the CagA^–^ group than in the CagA^+^ group (Figure 4A).

To further determine the function, gastric CX3CR1^+^CD4^+^ T cells were sorted and RNA was extracted for cytokine detection. We found that IFN-γ, IL-17A, and IL-4 levels increased significantly after *H. pylori* infection. The higher levels of IFN-γ and IL-17A in mucosal CX3CR1^+^CD4^+^ T cells were detected in the CagA^–^ group than in the CagA^+^ group (Figure 4B). These data indicated that CagA^–^*H. pylori* induced more functional CX3CR1^+^CD4^+^ Tem cell response than CagA^+^*H. pylori*.

### Number of Gastric CX3CR1^+^CD4^+^ Tem Was Negatively Correlated With Histopathologic Damage Score and *Helicobacter pylori* Colonization in *Helicobacter pylori*-Infected Mice

**FIGURE 5:**
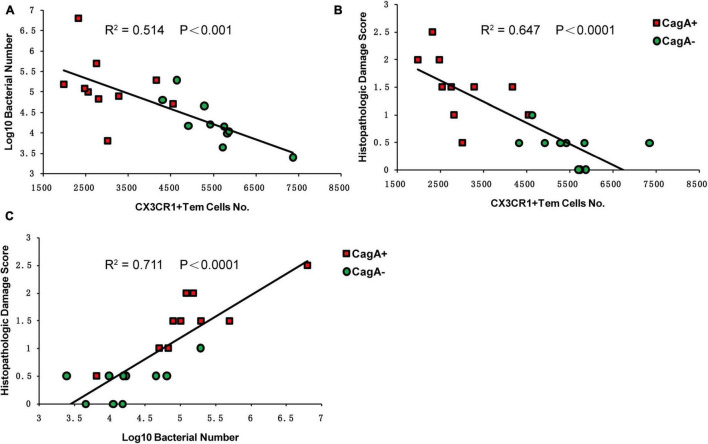
Pearson correlation analysis of CX3CR1^+^CD4^+^ Tem cell number, histopathologic damage score, and *H. pylori* colonization level in mice stomachs. Four weeks post *H. pylori* infection, CX3CR1^+^CD4^+^ Tem (CD3^+^CD4^+^CX3CR1^+^CD44^+^CD69^–^CCR7^–^) cell population, histopathologic damage score, and *H. pylori* colonization in the mice stomachs were measured. **(A)** Pearson correlation analysis of *H. pylori* colonization and CX3CR1^+^CD4^+^ Tem population. **(B)** Pearson correlation analysis of the histopathologic damage score and CX3CR1^+^CD4^+^ Tem population. **(C)** Pearson correlation analysis of the histopathologic damage score and *H. pylori* colonization. *N* = 20, the values of *R*^2^ and *P* are shown. Results are representative of three separate experiments.

Effector memory T cells are usually regarded as protective T cell responses (Hansen et al., 2009; Roestenberg et al., 2011). Our data also showed that more CX3CR1^+^CD4^+^ Tem cells were recruited to the stomach in the CagA^–^ group than in the CagA^+^ group. Thus, to determine the function of CX3CR1^+^CD4^+^ Tem cells, we performed a Pearson correlation analysis between each pair among the CX3CR1^+^CD4^+^ Tem cell number, *H. pylori* colonization level, and histopathologic damage score. We found that the CX3CR1^+^CD4^+^ Tem cell population was negatively correlated with *H. pylori* colonization level and histopathologic damage score (Figure 5). The *H. pylori* colonization level and the histopathologic damage score were positively correlated with each other (Figure 5). These data indicated that CX3CR1^+^CD4^+^ Tem cells in the mouse stomach may play a protective role in clearing *H. pylori*.

## Discussion

*Helicobacter pylori* is a Gram-negative bacterium in the stomach that infects half of the world’s population (Del Giudice et al., 2001). *H. pylori* infection is a key factor in the etiology of various gastric disorders, ranging from chronic gastritis without clinical symptoms to peptic ulceration and even gastric carcinoma (Suerbaum and Michetti, 2002). *H. pylori* virulence factor CagA, the most extensively studied virulence factor, is a 120–145 kDa protein encoded by the 40-kb cag pathogenicity island (PAI). It has been reported that approximately 60% of *H. pylori* strains isolated in Western countries carry cag PAI and almost all East Asian isolates are cag PAI-positive (Hatakeyama and Higashi, 2005). CagA can be injected directly from the bacterium into bacterium-attached gastric epithelial cells *via* T4SS (Schuelein et al., 2011). After being injected into host cells, CagA is able to activate SHP-2 tyrosine phosphatase (Hayashi et al., 2017) and interact with the human tumor suppressor apoptosis-stimulating protein of p53-2 (ASPP2) (Buti et al., 2011; Nesic et al., 2014), which favors *H. pylori* colonization (Buti et al., 2020). In this study, higher bacterial colonization was detected in the CagA^+^*H. pylori* infection group than in the CagA^–^*H. pylori* group.

Cytotoxin-associated gene A can upregulate NF-κB transcriptional activity and promote inflammation (Yamaoka, 2010). In this study, we found higher histopathological damage scores in CagA^+^ strain-infected mice than in CagA^–^ strain-infected mice (Figure 1). Inflammatory cells comprise neutrophils, eosinophils, lymphocytes, and other cells. Lymphocytes, particularly CD4^+^ T cells, are vital during *H. pylori* infection (Chen et al., 2013). In addition to CD4^+^ T cells, a number of CD8^+^ T cells can be observed in the stomachs of *H. pylori*-infected mice (Tan et al., 2008; Becher et al., 2010; Ruiz et al., 2012). The effects of CagA on inflammatory cells, including CD4^+^ and CD8^+^ T cells, should be different. These may be the reasons why higher histopathological damage scores accompanied by fewer CD4^+^ T cells were detected in CagA^+^ strain-infected mice than in CagA^–^ strain-infected mice (Figure 1C). In addition, the effects of CagA on T cells remain unclear. CagA and peptides from CagA have been confirmed to induce gastric/mesenteric lymph node and CD4^+^ T cells effectively secrete IFN-γ, which sensitizes mice to preneoplastic immunopathology rather than protecting them from *H. pylori* infection (Arnold et al., 2011). However, phytohemagglutinin-induced T cell proliferation can be inhibited by CagA (Paziak-Domanska et al., 2000). Bagheri et al. (2017) counted CD4^+^ T cells by immunohistochemical staining of mucosal biopsies from areas of antral gastric mucosa of patients with *H. pylori* infection. Their data showed that more CD4^+^ T cells were detected in mucosal biopsies from CagA^+^*H. pylori*-infected patients than in mucosal biopsies from CagA^–^*H. pylori*-infected patients (Bagheri et al., 2017). However, CagA has been shown to promote T cell death in a Fas-dependent manner (Larussa et al., 2015). In this study, we measured CD4^+^ T cells in the whole stomach by digesting the stomach into a single-cell suspension and found that mucosal CD4^+^ T cells increased post *H. pylori* infection and CagA^–^*H. pylori* recruited more CD4^+^ T cells in the stomach mucosa than CagA^+^ *H. pylori*.

Primed lymphocytes must migrate to the infection site to execute their function. Chemokines and their corresponding receptors play essential roles in CD4^+^ T cell recruitment. To confirm why more CD4^+^ T cells migrate to the gastric mucosa in CagA^–^*H. pylori*-infected mice than to the gastric mucosa in CagA^+^*H. pylori*-infected mice, we measured 24 chemokines in CagA^+^ or CagA^–^*H. pylori*-infected mice and found that compared with those in the CagA^+^ group, higher concentrations of CCL3 and CX3CL1 were detected in the CagA^–^ group and the concentration of CX3CL1 was much higher than that of CCL3 (Figure 2). The *in vitro* stimulation of gastric epithelial cells by *H. pylori* strains further confirmed that chemokine secretion can be affected by CagA^+^/CagA^–^ strains, particularly CX3CL1 (Figures 3C,D). CX3CL1 is primarily expressed in endothelial cells, which potently chemoattracts T cells and monocytes through the receptor CX3CR1. Therefore, in this study, CX3CR1 expression in mucosal lymphocytes was determined. We found that the number of CX3CR1^+^CD4^+^ T cells increased significantly after *H. pylori* infection. Moreover, the CagA^–^*H. pylori* strain induced more CX3CR1^+^CD4^+^ T cells in stomachs than the CagA^+^*H. pylori* strain (Figure 2B). In the chemotaxis assay, gastric CD4^+^ T cell migration to CX3CL1 was blocked by the CX3CL1 and CX3CR1 antibodies (Figure 3B). These results suggest that CagA^+^/CagA^–^ strains affect the recruitment of gastric CX3CR1^+^CD4^+^ T cells by changing CX3CL1 expression.

Memory T cells are a subset of “experienced” infection- or cancer-fighting T cells that have previously encountered and responded to their cognate antigen during a prior infection, encounter with cancer, or previous vaccination. They show greatly improved immune efficacy with a rapid and strong immune response. Until now, memory T cell populations have been considered to comprise Tcm, which are restricted to the secondary lymphoid tissues and blood; Tem, which broadly migrate between peripheral tissues, blood, and spleen; and Trm, which occupy tissues without recirculation (Tan et al., 2008). To phenotype the increased number of CX3CR1^+^CD4^+^ T cells in CagA^–^*H. pylori*-infected mice, we detected a series of memory cell markers and found that CX3CR1^+^CD4^+^ T cells were primarily CD44^+^CD69^–^CCR7^–^, a Tem phenotype, indicating that CX3CR1^+^CD4^+^ T cells were Tem cells (Figure 4A). Tem has been demonstrated to provide long-term protection against pathogenic infections. Hansen et al. (2009) reported that Tem responses were associated with the protection of rhesus monkeys from mucosal simian immunodeficiency virus challenge. Roestenberg et al.’s (2011) research also showed the long-term persistence of Tem responses in protected volunteers. In this study, we found that the number of CX3CR1^+^CD4^+^ Tem cells increased significantly after *H. pylori* infection, and these cells were detected in the CagA^–^*H. pylori* group (Figure 4). Thus, we wondered whether the increased number of CX3CR1^+^CD4^+^ Tem cells account for the low level of *H. pylori* colonization in CagA^–^*H. pylori*-infected mice. Pearson correlation analysis showed that the CX3CR1^+^CD4^+^ Tem cell number was negatively correlated with the *H. pylori* colonization level and the histopathologic damage score (Figure 5). Thus, CX3CR1^+^CD4^+^ Tem cells reducing *H. pylori* colonization and decreasing pathological damage may represent a possible mechanism. However, more work is needed to reveal the underlying mechanisms of Tem function.

*Helicobacter pylori*-associated gastric disorders can be derived from many factors, such as number and invasiveness of *H. pylori*, corresponding immune responses, including protective responses to clear *H. pylori* and inflammatory responses to cause immunopathological injury, and a variety of patient HLA, among others (Kusters et al., 2006). These factors also interact in various ways with each other, which are very complex. Minimally, however, we provide insight into the mechanism by which CagA^–^*H. pylori* causes less pathological damage than CagA^+^*H. pylori*.

BALB/c mice were used in the present study, considering that they contain two classical MHC-II molecules (I-A and I-E), but only the I-A allele exists in C57BL/6 mice. There should be no I-E-restricted CD4^+^ T cell responses in C57BL/6 mice. In this study, we focused on CD4^+^ T cells, despite no MHC restrictions. To avoid missing the I-E-restricted Hp-specific CD4^+^ T cell subsets artificially, BALB/c mice were chosen instead of C57BL/6 mice. Unfortunately, our conclusion has not been validated using knockout mice or other mouse strains. This is a limitation of our study. We are pushing the research forward, including obtaining more evidence from human subjects.

Overall, from our research, we found that more CD4^+^ Tem cells were recruited to the stomach mucosa of mice infected with CagA^–^*H. pylori* than to the stomach mucosa of mice infected with CagA^+^*H. pylori*. CD4^+^ Tem cell recruitment occurred primarily through the CX3CL1/CX3CR1 pathway, and the higher secretion of CX3CL1 was elicited by gastric epithelial cells infected with CagA^–^*H. pylori* than by the cells infected with CagA^+^ *H. pylori*. Although the gastric Tem cell number was negatively correlated with the bacterial colonization level in *H. pylori*-infected mice, this population cannot completely protect against *H. pylori* infection. *H. pylori* can reside in the gastric mucosa, causing chronic and persistent infection in mice and humans. The exact functions and mechanisms of CX3CR^+^CD4^+^ Tem cells remain unclear, and further evidence is warranted in the future.

## Data Availability Statement

The original contributions presented in the study are included in the article/Supplementary Material, further inquiries can be directed to the corresponding authors.

## Ethics Statement

The animal study wasprotect reviewed and approved by the Medicine Animal Care Committee of the Third Military Medical University.

## Author Contributions

CW and BL conceived theprotect study. HS, TH, and YW performed the experiments. HS, BL, and CW analyzed the data. HY, JN, and ZZ prepared the material. XD and CW provided the facility. BL and CW wrote the manuscript with the assistance of other authors. All authors contributed to the article and approved the submitted version.

## Conflict of Interest

The authors declare that the research was conducted in the absence of any commercial or financial relationships that could be construed as a potential conflict of interest.

## Publisher’s Note

All claims expressed in this article are solely those of the authors and do not necessarily represent those of their affiliated organizations, or those of the publisher, the editors and the reviewers. Any product that may be evaluated in this article, or claim that may be made by its manufacturer, is not guaranteed or endorsed by the publisher.

## Supplementary Material

Supplementary Figure 1 Chemokine receptors on gastric CD4^+^ T cells. Mice were infected with *H. pylori* strain NCTC11637 (CagA^+^) or its isogenic mutant ΔcagA NCTC11637 (CagA^–^) *via* intragastric administration. Four weeks later, the stomachs were harvested, and chemokine receptors expressed on gastric mucosal CD4^+^ T cells were determined. Data are shown as mean ± SD. *N* = 5, **p* < 0.05, ^**^*p* < 0.01, and ^***^*p* < 0.001.
