# Supplementary figures and images for: Cytotoxin-Associated Gene A-Negative Helicobacter pylori Promotes Gastric Mucosal CX3CR1+CD4+ Effector Memory T Cell Recruitment in Mice
Source: Front Microbiol. 2022 Jan 27;13:813774. doi: 10.3389/fmicb.2022.813774 (PMC8829513; doi:10.3389/fmicb.2022.813774)

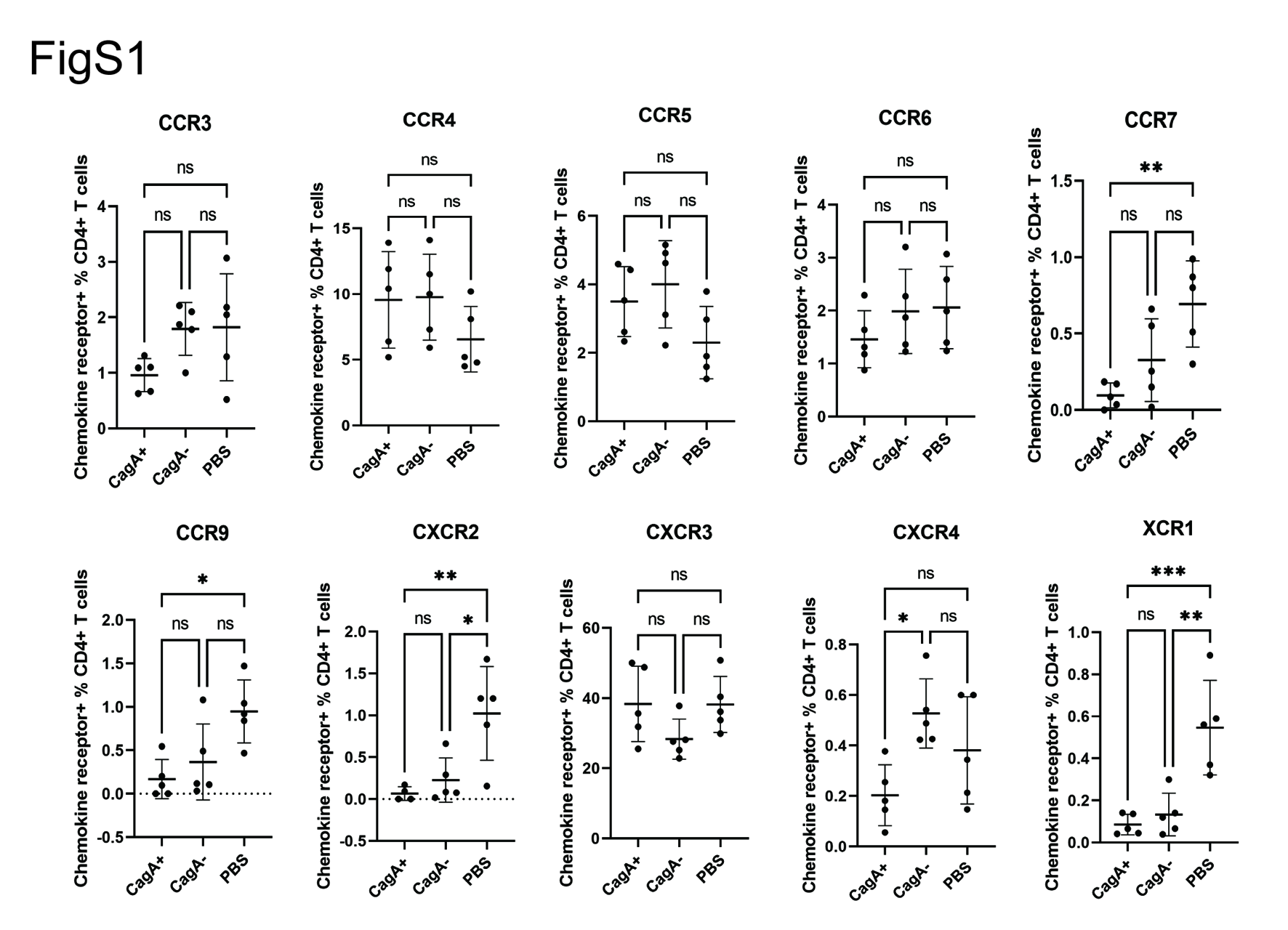

Supplement: Supplementary Figure 1 — Chemokine receptors on gastric CD4+ T cells. Mice were infected with H. pylori strain NCTC11637 (CagA+) or its isogenic mutant ΔcagA NCTC11637 (CagA–) via intragastric administration. Four weeks later, the stomachs were harvested, and chemokine receptors expressed on gastric mucosal CD4+ T cells were determined. Data are shown as mean ± SD. N = 5, *p < 0.05, **p < 0.01, and ***p < 0.001. [file Image_1.TIF]
